# Integrating miRNA and mRNA Expression Profiling Uncovers miRNAs Underlying Fat Deposition in Sheep

**DOI:** 10.1155/2017/1857580

**Published:** 2017-02-15

**Authors:** Guangxian Zhou, Xiaolong Wang, Chao Yuan, Danju Kang, Xiaochun Xu, Jiping Zhou, Rongqing Geng, Yuxin Yang, Zhaoxia Yang, Yulin Chen

**Affiliations:** ^1^College of Animal Science and Technology, Northwest A&F University, Yangling, China; ^2^Lanzhou Institute of Husbandry and Pharmaceutical Sciences of CAAS, Lanzhou, China; ^3^College of Biology Sciences and Engineering, Beifang University of Nationalities, Yinchuan, Ningxia 750021, China; ^4^Qinghai Academy of Animal Science and Veterinary Medicine, Xining, China; ^5^College of Pharmacy, Yancheng Teachers University, Yancheng, China

## Abstract

MicroRNAs (miRNAs) are endogenous, noncoding RNAs that regulate various biological processes including adipogenesis and fat metabolism. Here, we adopted a deep sequencing approach to determine the identity and abundance of miRNAs involved in fat deposition in adipose tissues from fat-tailed (Kazakhstan sheep, KS) and thin-tailed (Tibetan sheep, TS) sheep breeds. By comparing HiSeq data of these two breeds, 539 miRNAs were shared in both breeds, whereas 179 and 97 miRNAs were uniquely expressed in KS and TS, respectively. We also identified 35 miRNAs that are considered to be putative novel miRNAs. The integration of miRNA-mRNA analysis revealed that miRNA-associated targets were mainly involved in the gene ontology (GO) biological processes concerning cellular process and metabolic process, and miRNAs play critical roles in fat deposition through their ability to regulate fundamental pathways. These pathways included the MAPK signaling pathway, FoxO and Wnt signaling pathway, and focal adhesion. Taken together, our results define miRNA expression signatures that may contribute to fat deposition and lipid metabolism in sheep.

## 1. Introduction

Adipocytes are cells that form and store fat globules in the body. Adipose tissue deposits are located in different regions of the body, primarily in the form of subcutaneous fat or intramuscular fat in domestic animals. Though the volume of adipocytes varies from breed to breed and is influenced by diet, genetic factors are considered major determinants for the formation of adipocytes. Fat-tailed sheep, which exhibit distinctive large tails and hindquarters, comprise approximately 25% of the world's sheep population [1] and are raised commercially for meat, milk, fat, or wool production. Fat-tail is regarded as an adaptive response to the harsh challenges of desert life and the fat deposits provide a valuable energy reservation during drought seasons. The Kazakhstan sheep (KS, fat-tail breed) and Tibetan sheep (TS, thin-tail breed) are two native breeds raised in the extremely arid regions of western China, exhibiting distinct phenotypes of tails as previously described [2].

MicroRNAs (miRNAs) are a set of small noncoding RNAs (~22 nt) that bind to partially complementary sequences on target mRNAs resulting in posttranscriptional regulation of gene expression [3]. MicroRNAs play key roles in regulating numerous biological processes in developmental, cell differentiation, and disease processes. These include adipogenesis and obesity in humans [4–7], as well as lipid metabolism and fat deposition in livestock [8, 9]. Additionally, evidence suggests that miRNAs regulate the formation of adipose tissue. For instance, miR-378 was strongly associated with the thickness of back fat in beef cattle [10]. In recent years, next generation sequencing has been widely used to explore molecular mechanism of adipogenesis in sheep. However, miRNA-mRNA correlation analysis relating to adipogenesis in sheep has not been examined as in other livestock species [11, 12]. This research provides an understanding of the process in sheep and contributes to the understanding of obesity and related diseases.

We have previously described specific changes of transcriptomic differences with respect to adipose tissues from the representative KS and TS breeds and identified a list of candidate genes underlying the phenotypic differences of fat/thin tails in sheep [2]. In this study, we conducted miRNA sequencing using the same tissues, in attempt to elucidate the roles of miRNAs in the presentation of these two extreme phenotypes. We further employed a systems biology approach to examine the correlation between miRNA and mRNA expression data to identify potential miRNA-associated target genes. Here, we have implicated interactions from particular cellular processes such as the MAPK signaling pathway, FoxO and Wnt signaling pathway, and focal adhesion, gaining insights into the potential roles of miRNAs in the regulation of these pivotal pathways.

## 2. Materials and Methods

### 2.1. Ethics Statement

The experiments were conducted following the guidelines of the Animal Ethics Committee at Northwest A&F University under the document 2011-31101684. The sampling procedures complied with the “Guidelines on Ethical Treatment of Experimental Animals” (2006) Number 398 set by the Ministry of Science and Technology, China.

### 2.2. Animals and Phenotypes

Six adult individuals (three males and three females, two years old) from KS and TS breeds were randomly selected, respectively, and any two or more individuals with a traceable phylogenetic relationship were avoided in the sampling process. Adipose tissue biopsies were collected from the tails and stored as previously described [2].

### 2.3. Small RNA Library Construction and Sequencing

Total RNA from the mixed adipose tissues of six adult sheep was isolated using the RNAiso plus kit (TaKaRa, Dalian, China) according to the manufacturer's protocol. The small RNA libraries were prepared following Illumina® TruSeq™ Small RNA Sample Preparation protocol. The small RNA libraries were sequenced using an Illumina/Solexa 1 G Genome Analyzer System (BGI, Shenzhen). The sequencing reads were deposited to the Sequence Read Archive, under accession code SRP093866.

### 2.4. Expression Profiling

The raw miRNA sequencing reads were filtered and only the unique mapping reads were used for subsequent bioinformatics analysis, such as annotation and gene expression. The expression of miRNA in two samples (TS and KS) was normalized by transcripts per million (TPM) as previous description [13]. After normalization, the expression value of a miRNA, more than one in the two samples, was kept for the differential expression analysis, and we revised 0 value to be 0.01 when we calculated fold change of the two breeds [14]. The differentially expressed genes (DEGs) were determined according to its absolute fold change (log_2_(TS/KS)) of ≧1 and *P* value of ≦0.05. Scatter plots were used to demonstrate differentially expressed miRNA between the two sheep phenotypes.

### 2.5. Quantitative RT-PCR

Total RNA from the mixed sheep tails (six from KS and six from TS) was isolated using a miRNA extraction kit (CWBIO, Beijing, China). First strand cDNA was synthesised with reference to RevertAid First Strand cDNA Synthesis kit (Thermo Scientific #K1622, Fermentas) manufacturer's instructions, using special stem-loop RT primers [15] (Additional file 1 in Supplementary Material available online at https://doi.org/10.1155/2017/1857580). PCR-iQ5 system (Bio-Rad, Hercules, CA, USA) was used to analyse miRNA genes expression level. The reaction mixture volume is 25 *μ*L, containing 2 *μ*L of cDNA (25 ng), 12.5 *μ*L SYBR Premix Ex TaqTM II (TaKaRa, Dalian, China), 1.0 *μ*L specific forward primer, 1 *μ*L universal primer, and 8.5 *μ*L water, the reaction program referenced to previous description [14]. U6 snRNA was used as an endogenous control. All reactions were performed in triplicate, and 2^−ΔΔCt^ method was used to analyse the data. *t*-tests (parametric test, unpaired test) was used to determine significance of the results using SPSS21. Graphpad Prism 6 was used to plot the graphs. All data were presented as mean ± SD and values of *P* < 0.05 were considered statistically significant.

### 2.6. mRNA Profiling Bioinformatics

The sequences of reads with poly(A) tracts were deposited to the Sequence Read Archive, under accession code SRP093860. The FastQC package was used to assess the quality of raw reads and then mapped to the sheep genome assembly Oar_v3.1 (GCA_000298735.1) using TopHat2 [16]. Quality control was performed on the two samples. The high-quality (HQ) clean reads were presented after a series of procedures (remove adapter, ploy N, and low-quality reads). All the following analyses were based on the HQ clean reads. The unannotated HQ clean reads were assembled by Cufflinks [17]. Orthonormal expression levels were calculated as FPKM (Fragments Per Kilobase of transcript per Million mapped reads) [18], and FPKM distribution of the samples was presented as gene's coverage. The edgeR package for bioconductor was applied to identify differentially expressed genes between TS and KS [19]. Genes were defined as differentially expressed with the threshold FDR < 0.05 and |log_2_⁡(fold change)| > 1.

### 2.7. Integration Analysis of miRNA and mRNA Sequencing Data

MicroRNA-mRNA integration analysis was performed on the mRNAs that were differentially expressed between the KS and TS breeds in our previous study [2], and the miRNAs were identified and classified according to the miRNA family again, in order to meet the current study. Individual miRNAs determined to be differentially expressed were submitted to each of the three databases ((RNAhybridv2.1.2)+svm_light(v6.01), Miranda(v3.3a), and TargetScan(Version: 7.0)) to determine the predicted mRNA targets, and the parameter was taken as the default. We selected only mRNA targets that were differentially expressed in the same samples. Next, the correlation between the expression levels of each miRNA and any of the predicted, differentially expressed target mRNA from any of the three databases was computed. According to the analysis results and the prior research, we chose miRNA-125a-5p and one of the target genes (ESRR*α*) for validation analysis.

### 2.8. Functional Enrichment of Target Genes

For each miRNA with a significant association with its set of mRNA targets (based on the gene set enrichment test), the GO and KEGG terms were extracted for all of its mRNA targets. We reveal the functions significantly related to predicted target gene candidates of miRNAs used gene ontology (GO) analysis.

The primary pathways of the candidate target genes were determined by KEGG pathway analysis. The formula used to calculate this is the same as that used in the GO analysis. Genes with *P* < 0.05 are considered as significantly enriched in target gene candidates. The KEGG indicates the main pathways in which the candidate genes are involved.

## 3. Results

### 3.1. Small RNA Diversity in Sheep Adipose Tissues

**Figure 1:**
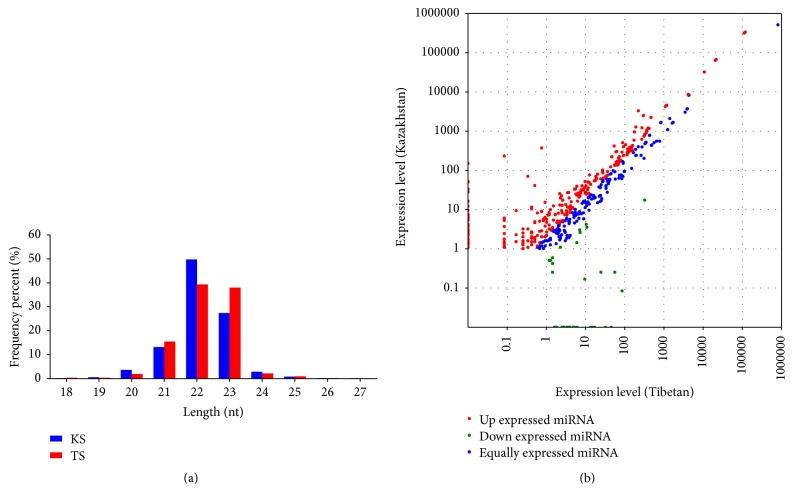
(a) Length distribution of the clean reads based on total abundance and distinct sequences. (b) Differential expression of miRNAs between the adipose tissues from KS and TS breed. Each point represents a single miRNA. The *X* and *Y* axes show the expression level of miRNAs in these two samples, respectively. Red points represent miRNAs with a ratio > 2, blue points represent miRNAs with 1/2 < ratio ≦ 2, and green points represent miRNAs with ratio ≦ 1/2; ratio = normalized expression in treatment/normalized expression in control.

The sequencing of two small RNA libraries from the KS and TS yielded 12 M counts of sequencing reads, respectively (Table 1). All of the clean reads were aligned with the sheep genome (Oar_v3.1) sequence using SOAP software. The length distribution and the distribution of miRNAs on the genome were analysed. The high-quality reads in both groups exhibited the canonical size range distribution that is common to mammalian miRNAs [3] (Figure 1(a)). The vast majority of the reads were approximately 21~23 nucleotides (nt) in length, and ~22 nt reads accounted for 50% and 39% for each of high-quality reads from the KS and TS breeds, respectively. This is in agreement with previous founding in other adipose tissues [20]. These results demonstrated the high reliability of using the small RNA-sequencing approach to obtain miRNA reads for further studies.

There were 8,000,311 (67.53%) and 6,720,288 (56.68%) reads matched to the sheep genome sequence in KS and TS libraries, respectively. All clean reads were classified and annotated by tag2annotation software (developed by BGI), aligned against the Rfam database (Ver. 10.1) and the miRBase (Ver. 19.0). There were 11,599,356 and 11,603,988 total conserved miRNAs reads, and 0.77% and 0.81% of clean reads were identified as potential novel miRNAs from KS and TS libraries, respectively, and more information was given in the additional file (Additional file 2).

### 3.2. Differential miRNA Expression in Sheep Adipose Tissues

To identify conserved miRNAs in sheep, all sRNA sequences were mapped to known miRNAs in the miRBase 19.0 database. We presented a total of 815 miRNAs found in abundance in adipose tissues of sheep (Additional file 3). Of 815 identified miRNAs, 539 miRNAs were expressed in both breeds, whereas 179 and 97 miRNAs were specifically expressed in KS and TS, respectively. The top ten abundant miRNAs from KS, TS, and both groups are listed in Table 2. Of the shared miRNAs, 93 were upregulated and 33 were downregulated in the adult adipose tissues of sheep (Figure 1(b)).

**Figure 2:**
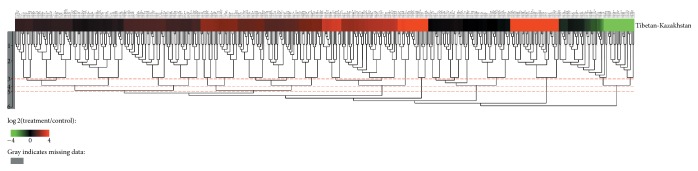
Heat map of miRNAs differentially expressed in sheep adipose tissues. Red indicates that the miRNA has a higher expression level in the treatment samples; green indicates that the miRNA has a higher expression in the control samples and gray indicates that the miRNA has no expression in at least one sample. Each row in the figure represents one miRNA, and each column shows one sample pair. Each cell shows the differential expression of a miRNA in one sample pair.

We next analysed the differentially expressed miRNAs in the two breeds. One hundred and seventy-five miRNAs exhibited similar expression levels (nonsignificant), 33 miRNAs showed significantly reduced expression (*P* < 0.05) and 276 miRNAs showed an increase expression (*P* < 0.05) in KS, compared to TS (Figure 1(b)). MicroRNAs with similar expression patterns in different sample pairs were clustered together. Clustering analysis was based on the sample difference model by using Cluster software, and the results were viewed with Java Treeview. All differentially expressed miRNAs clustered together after six rounds of clustering (Figure 2).

Novel miRNAs could be predicted through the characteristic hairpin structure of miRNA precursors. We predicted novel miRNAs by exploring secondary structure, the Dicer cleavage site, and the minimum free energy of the unannotated small RNA reads, which could be mapped to sheep genome sequences by using Mireap software. In total, 186,755 unannotated sequences were used to predict novel miRNAs. Thirty-five potential novel miRNAs were identified; 7 miRNAs were expressed in the two samples, whereas 18 and 10 miRNAs were specifically expressed in KS and TS, respectively (Additional file 4).

### 3.3. Validation of miRNA by qPCR

**Figure 3:**
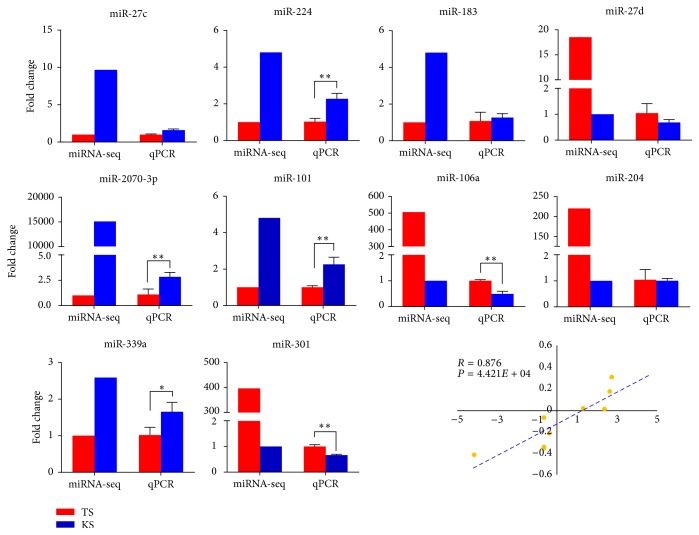
qPCR validation of miRNAs identified in sheep adipose tissues. Red represents TS and blue represents KS (0.01 < ^*∗*^*p* < 0.05, ^*∗∗*^*p* < 0.01).

In order to validate the reliability of miRNA expression data, quantitative RT-PCR was conducted to assess the expression of six upregulated miRNAs (miR-27c, miR-183, miR-224, miR-339a, miR-2070-3p, and miR-101) and four downregulated ones (miR-27d, miR-106a, miR-204, and miR-301) among the differentially expressed miRNAs, using adipose tissues from both KS and TS groups. U6 was used as an internal control. Stable miRNA expression levels in both KS and TS tissues correlated well between qPCR and miRNA-seq data (Figure 3).

### 3.4. Differential Expression of mRNAs in TS and KS Sheep Adipose Tissue

In total, 55,354,542 and 51,410,666 clean reads were obtained from the TS and KS adipose tissues, respectively. The percentages of reads containing HQ clean reads, adaptors, N, low-quality reads, poly A, and clean reads were calculated. Approximately 99% of the clean reads passed the filter (Additional file 5). After alignment by TopHat, 86.78% and 86.65% unique reads were found in Tibetan and Kazakhstan sheep, respectively (Additional file 6). We identified 15330 (76.13%) known genes and 458 novel genes in Tibetan sheep and 15162 (75.29%) known genes and 459 novel genes in Kazakhstan sheep. Those expression levels were quantified by FPKM ≥ 0.05 (Additional file 7).

**Figure 4:**
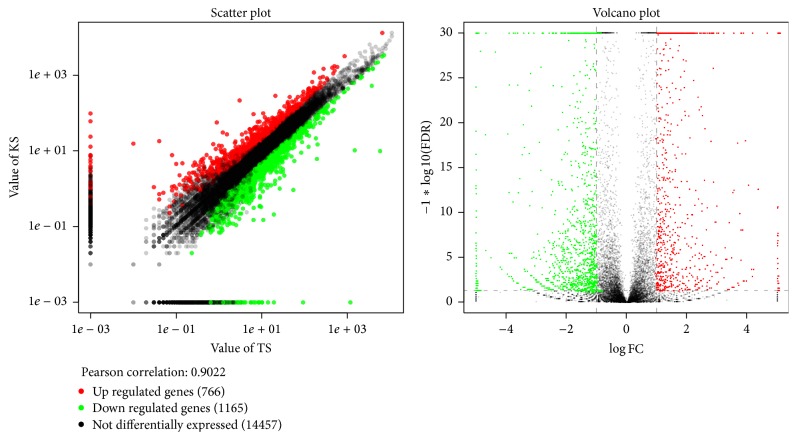
Differentially expressed gene between TS and KS DEGs. Red spots represented upregulated genes, and green spots indicated downregulated genes. Back spots represented genes that did not show obvious changes between the TS and KS.

DEGs were selected out by FDR < 0.05 and FPKM fold change (|log_2_⁡(KS/TS)|). Compared with KS, 1931 DEGs were identified. Seven hundred and sixty-six of these were upregulated expression, and 1165 were downregulated (Additional file 8). The scatter plot showed significant DEGs in the above comparisons between TS and KS (Figure 4).

### 3.5. GO and Pathway Enrichment Analyses of Differentially Expressed Genes

**Figure 5:**
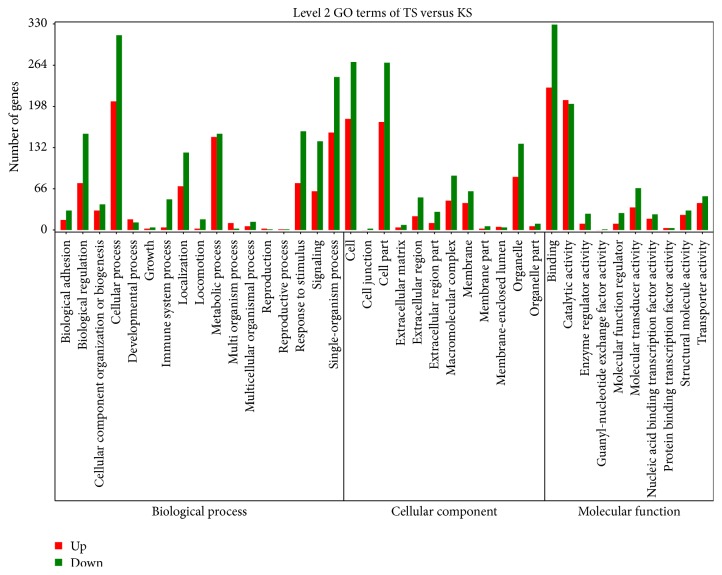
GO classification of DEG. The *x*-axis indicated the subcategories; the right *y*-axis indicated the number of DEG. The height of columns represented the percentage (number) of upregulated expression (red) and downregulated expression (green) genes.

To determine the function of differentially expressed genes, all of the DEGs in this study were mapped to terms in the GO database (http://www.geneontology.org/) using Blast2GO [21]. In order to assess the general functional characteristics of genes activity in sheep adipose tissues, we performed functional enrichment analysis of GO using DAVID software and pathway enrichment using specific KEGG terms [22]. A total of 1522 genes were categorized into the three main categories of GO classification.  Figure 5 shows the GO classification of DEGs.

**Figure 6:**
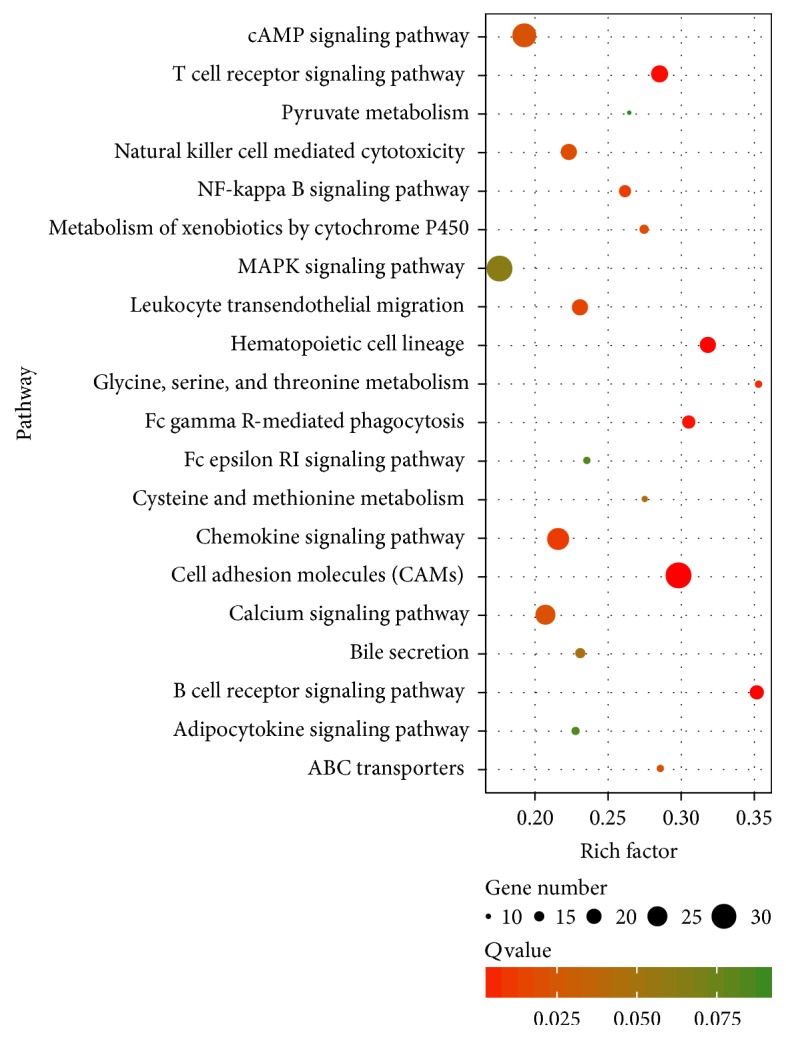
Top 20 of pathway enrichment. The circle size represented gene number. *Q*-value was shown by color gradient.

KEGG pathway annotation showed that 4899 genes and 537 DEGs genes were annotated for 209 biological functions (Additional file 9).  Figure 6 showed the top 20 of pathway enrichment. We found that several significantly overrepresented categories of GO biological processes were associated with fat metabolism and deposition. We found that the cAMP signaling pathway had a critical role in both adipogenesis and lipid partitioning in white adipose tissue and well-characterized mechanisms controlling adipocyte differentiation [23, 24].

### 3.6. Identification of Potential mRNA Targets of miRNAs

MicroRNA recognizes its target mRNA through binding to a seed sequence, which localizes on the 3′ untranslated regions (3′UTR) of target mRNAs [25]. In an attempt to determine potential genes whose mRNAs might be targeted by particular miRNAs, we conducted an integrated analysis between the expression of specific miRNA and the expression of all the predicted miRNA target genes (mRNA) using TargetScan. We identified potential miRNA target genes that are downregulated at the transcriptional level and are inversely correlated with the miRNA expression in the same KS and TS adipose tissues.  Table 3 lists the top 10 differentially expressed miRNAs and their target genes determined by miRNA-mRNA correlation analysis, and the details were presented in Additional file 10.

### 3.7. Validation of the Target Gene of miR-125a-5p

**Figure 7:**
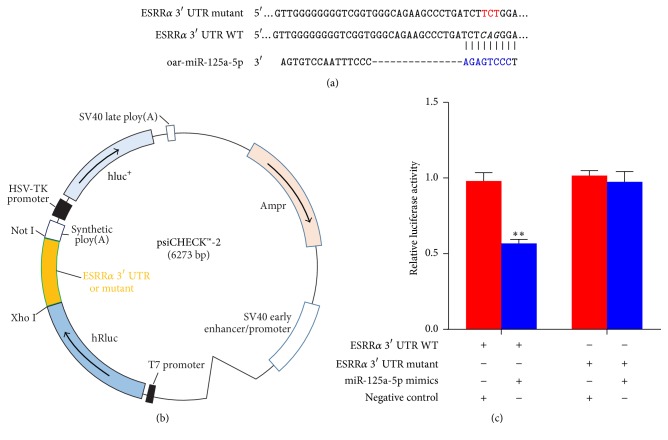
miR-125a-5p downregulates the expression of ESRR*α* by targeting its 3′ UTR site. (a) The pairing schematic of ESRR*α* 3′ UTR and miR-125a-5p. The nucleotide in blue represents “seed sequence” of miR-125a-5p, and the mutation nucleotides are presented in red. (b) The insertion site of ESRR*α* 3′ UTR or its mutation luciferase reporter vector map. (c) The two types were cotransfected with miR-125a-5p mimics (or negative control) into 293T cells. Luciferase assay was performed 48 h after transfection. Results are presented as relative luciferase activity (mean ± SD; *n* = 4; ANOVA; ^*∗∗*^*P* < 0.01).

For validation, we selected the a priori gene ESRR*α*, which inhibits preadipocyte differentiation [26, 27]. Using RNAhybrid (http://bibiserv.techfak.uni-bielefeld.de/rnahybrid) and miRanda (http://www.microrna.org) and combining the results of the analyses, we presumed that miR-125a-5p regulates ESRR*α* translation. MiR-125a-5p was found to directly target the ESRR*α* 3′ UTR sequence (Figure 7(a)). To verify this finding, the 3′ UTR containing miR-125a-5p targeted site was cloned and inserted into the psiCHECK™-2 reporter plasmid (psiCHECK™-2-ESRR*α*-3′UTR WT) and also the mutated 3′ UTR (psiCHECK™-2-ESRRa-3′UTR-mutant). The two types were cotransfected with the miR-125a-5p mimics or negative control sample into 293T cells, respectively, and luciferase activity was measured after 48 h later. The luciferase activity of the miR-125a-5p group was significantly lower than that of the NC group (*P* < 0.01), whereas the mutated ESRRa 3′ UTR exhibited increased luciferase expression (Figure 7(c)). Thus, the miR-125a-5p was confirmed as one of the ESRR*α* translation targeted regulation sequences and the primer sequences used for this are listed in Additional file 1.

### 3.8. Functional Enrichment Based on miRNA-Associated Target Genes

In order to assess the general functional characteristics of the miRNA-related activity in sheep adipose tissues, we selected the putative miRNA target genes derived from miRNA-mRNA correlation analysis and performed functional enrichment analysis of gene ontology (GO) using DAVID software and pathway enrichment using specific KEGG terms [22]. We found that several significantly overrepresented categories of GO biological processes were associated with biological functions, including environmental information processing, cellular processes, metabolism, and genetic information processing (Additional file 11). KEGG pathway annotation showed that 4,687 target genes were annotated for 226 biological functions (Additional file 12). Most of these genes were involved in metabolic processes, regulation of biological process, and signal transduction. Several of these KEGG terms were related to fat deposition and fatty acid metabolism, including MAPK signaling pathway, focal adhesion, Pyruvate metabolism, FoxO signaling pathway, and TNF signaling pathway. This suggests that miRNAs are functionally relevant to lipid metabolism and fat deposition processes.

## 4. Discussion

Next generation sequencing approaches have become powerful tools to predict and identify the novel genes and small RNAs of livestock species and other organisms. Compared with other meat-producing livestock such as cattle and pig, fewer studies have focused on the role of miRNAs in the process of fat deposition in sheep, though there are a few omics studies screening miRNAs in sheep skin [28] and muscles [29]. In an effort to gain insights into the molecular mechanisms underlying fat deposition of adipose tissue governed by miRNA regulation in a fat-tailed and a thin-tailed sheep breeds, we evaluated whole transcriptome profiles of miRNAs and mRNA expression on the same adipose tissue from sheep tails [2].

In the present study, we were able to discover 35 novel miRNAs which have eluded previous efforts and identify 239 miRNAs that have not been previously annotated in sheep. Our analysis detected 815 differentially expressed miRNAs between the fat deposits of the two sheep breeds used here. Not surprisingly, a proportion of the miRNAs was found to be differentially expressed in our study had been previously reported to play a role in adiposity, adipocyte development/differentiation, and other metabolic disturbances in livestock, as well as adiposity and related disease risk in humans. This further highlights the importance of miRNAs in adipose tissue development and metabolism. Previous studies have shown an increase in miR-143 during human and murine preadipocyte differentiation, and the following experiment verified that it inhibited preadipocyte differentiation [30–32]. miR-378 may promote bovine adipogenesis in white adipose tissue through targeting MAPK1 and PPAR*γ* [10]. The interactions demonstrated effects on the MAPK signaling pathway, focal adhesion, and Pyruvate metabolism pathways. miR-103 and miR-30 have been reported to enhance adipogenesis [32–35], while miR-27 and miR-138 could inhibit adipogenesis [36, 37]. miR-122, miR-370, and miR-378 have been demonstrated to have a key role in lipid metabolism [7, 38, 39]. miR-148a might modulate fat deposition by targeting MAPKAPK5, MAPK3, and MAP2K2, and miR-148 has been shown to affect obesity and modulates adipocyte differentiation [40, 41]. miR-125b-5p inhibited proliferation and promoted adipogenic differentiation in 3T3-L1 preadipocytes [42], and other studies have shown that miR-125b impaired brite adipocyte formation and function [43]. These reports are consistent with our findings reported.

In addition to microRNA expression profiling, we performed miRNA-mRNA integration analysis using the miRNA and mRNA expression data from corresponding tissues in distinct sheep breeds and determined a limited number of putative target genes for the differentially expressed miRNAs. The integration analysis removed a large portion of false positive miRNA target genes and increased the precision of our predictions [44]. Therefore, the identified candidates could be taken as reference genes to validate the differentially expressed miRNAs really affecting the mRNA expression levels during adiposeness. Here, we showed that miR-125a inhibits porcine preadipocytes differentiation by targeting ESRR*α* [26]. This kind of targeted effect also exists in sheep, so we speculate that the function of miR-125a in ovine preadipocytes is similar to that in porcine preadipocytes.

The DAVID gene annotation analysis of the miRNA-associated targets indicated that the GO biological processes were enriched to modification processes, metabolic processes, signal transduction, and regulation of biological processes. This demonstrates the distinct roles of adipocyte development in the tail areas as the reserves of energy, in response to external or internal environmental conditions during the dry and cold seasons. KEGG pathway annotation of their putative miRNA-related targets revealed that the MAPK signaling pathway, focal adhesion, Pyruvate metabolism, FoxO signaling pathway, and TNF signaling pathway contribute to the formation of sheep fat/thin-tails phenotypes. Various tail phenotypes are unique for sheep compared to other animals in the word, interestingly, a mass of adipose depot in the fat-tail sheep breed and less in the thin-tail sheep breed. In a manner, this phenomenon is a form of energy storage in the fight against extreme conditions, and the function of tail adipose tissue is similar to subcutaneous adipose tissue of other animals according to the enrich pathways, which is associated with metabolism risk [20, 45].

## 5. Conclusion

In conclusion, we identified a number of miRNAs that are differentially expressed between the fat-tailed and short-tailed sheep breeds. We further highlighted gene targets of related miRNAs that may be involved in regulating fat deposition and adiposeness, in sheep and other livestock. This occurs via the key signaling pathways including focal adhesion, Pyruvate metabolism, and the MAPK, FoxO, and TNF signaling pathway. Further studies are needed to verify the correlation between key miRNAs and their target genes by in vitro approach and elucidate the functional impacts that miRNAs serve during adiposeness. Our results also provide evidence for the interaction of miRNAs and genes in the regulation of obesity and metabolic syndromes, which suggests that this may serve as an animal model for human' obesity and metabolic syndromes researches.

## Supplementary Material

Additional file 1: Primer pairs used for this study. Additional file 2: The distribution of miRNAs in TS and KS separately. Additional file 3: The finding miRNAs list. Additional file 4: Novel miRNAs identified in sheep adipose tissues. Additional file 5: Filter Information for Reads. Additional file 6: Mapping Information. Additional file 7: Gene expression details for KS and TS. Additional file 8: List of the identified DEGs in fat tissues transcriptome in sheep. Additional file 9: TS vs KS DEGs genes pathway enrichment. Additional file 10: The detail correlation of miRNA-mRNA. Additional file 11: Predicated target genes GO Enrichment (Biological Process). Additional file 12: Predicated target genes pathway enrichment.

## Figures and Tables

**Table 1:** Summary of total small RNA tags by Solexa sequencing.

Type	KS		TS	
	Counts	Percentage (%)	Counts	Percentage (%)
total_reads	12,000,000		12,000,000	
high_quality	11,977,968	100	11,973,801	100
3′adapter_null	54,706	0.46	61,670	0.52
insert_null	4,294	0.04	5,122	0.04
5′adapter_contaminants	5,406	0.05	5,610	0.05
smaller_than_18nt	66,241	0.55	45,410	0.38
polyA	4	0.00	3	0.00
clean_reads	11,847,317	98.91	11,855,986	99.02

**Table 2:** Highly expressed miRNAs (top 10) in sheep adipose tissues.

In fat-tailed tissue (KS)		In thin-tailed tissue (TS)		In both tissues	
miRNA	*P* value	miRNA	*P* value	miRNA	*P* value
miR-2070-3p	0	miR-4792	3.55*E* − 18	miR-25-3p	2.07*E* − 01
miR-222	2.86*E* − 189	miR-106a	8.87*E* − 19	miR-25	1.09*E* − 02
miR-502-3p	1.24*E* − 179	miR-3649	4.44*E* − 19	miR-10a-5p	2.43*E* − 18
miR-7446-3p	8.35*E* − 122	miR-4152-5p	2.17*E* − 22	miR-10	3.94*E* − 18
miR-6724-5p	4.92*E* − 104	miR-125a-3p	3.34*E* − 302	miR-10a	1.77*E* − 23
miR-126	2.53*E* − 101	miR-4492	5.80*E* − 48	miR-92a-3p	1.01*E* − 26
miR-378e	0	miR-993-5p	5.57*E* − 54	miR-10-5p	5.49*E* − 72
miR-30c	9.26*E* − 60	miR-7930-3p	3.35*E* − 61	miR-92b-3p	4.72*E* − 29
miR-193	3.71*E* − 59	miR-7475-5p	2.33*E* − 115	let-7	0
miR-2312	2.13*E* − 44	miR-6238	5.41*E* − 162	miR-486-5p	0

**Table 3:** Lists of miRNA and their targets with functions related to adipogenesis and/or fat metabolism.

miRNA	Target numbers	Partial target genes
miR-2070-3p	1352	SH3D21, BCL7C, ACTR3B, EPC1
miR-222	624	RGS6, HMG20A, RBM15, NFE2
miR-502-3p	462	CRTC1, FGD1, CCL8, STARD8
miR-6238	391	Mcph1, PDZK1
miR-7446-3p	414	KLF13, SIAH2, TUB
miR-7475-5p	517	LDB1, DVL3, PEG3, LRP1, LATS2, EFHD2
miR-125a-5p	2246	ESRR*α*, SENP2, BCL2L12, SREBP-1, ABCA2, NNMT
miR-126	2438	TNKS2, PTPRU, RGS14, NAP1L5
miR-378e	1044	IGF1R, CACNB2, RASIP1, API5, SCD5, SLC25A29
miR-7930-3p	1793	CABIN1, PCDHA2, PLXNA4
